# The “Movie Theater” Study: Acute Cardiometabolic Effects of a Cinema-Style Meal

**DOI:** 10.3390/metabo16020139

**Published:** 2026-02-18

**Authors:** Jenna K. Schifferer, Alexis R. Quirk, Morgan E. Higgins, Sarah E. Fruit, Natalie G. Keirns, Bryant H. Keirns

**Affiliations:** Department of Nutrition and Health Science, Ball State University, Muncie, IN 47303, USA

**Keywords:** refined sugar, hyperglycemia, carbohydrate metabolism, vascular function, intestinal permeability

## Abstract

**Background/Objectives**: Meals eaten at movie theaters may have acute, negative health effects due to high refined sugar and moderate sodium content. We aimed to characterize the cardiometabolic response to movie-theater-style meals independently (fasting) and after high-fat meal consumption. **Methods**: Participants (N = 10; 5M/5F; 18–45 y) completed two meal trials (randomized). At both trials, participants ate a movie-theater-style meal (popcorn, candy, and soda; 884 kcal, 150 g sugar, and 700 mg sodium). At one trial, the movie theater meal was consumed while fasting (Fasting Trial). At the other trial, a high-fat meal (820 kcal; 56 g fat) was consumed 3.5 h prior to the movie theater meal (Fed Trial). Blood was collected (0, 0.5, 1, 2, 3, and 4 h) and endothelial function (i.e., flow-mediated dilation or FMD) was assessed (0, 2, and 4 h) at both trials. Serum metabolic markers (glucose, insulin, triglycerides, and HDL-C) and biomarkers of intestinal permeability (sCD14 and LBP) were measured. Mixed-model ANOVAs (meal × time) and change scores (Δ) were used to compare responses between trials. **Results**: At both trials, glucose, insulin, and triglycerides increased, while HDL-C decreased (*p*_time_’s ≤ 0.05). ΔInsulin (*p* = 0.02), but not Δglucose, was higher at Fasting versus Fed. Peak glucose (range = 86–178 mg/dL) and insulin (range = 28.3–307.6 mU/L) were highly variable between participants across trials. Absolute and percent FMD tended to decrease, regardless of trial (*p*_time_’s ≥ 0.08). **Conclusions**: Overall, the movie theater meal impacted a number of cardiometabolic factors when consumed independently and after a high-fat meal, although there was notable inter-individual variability.

## 1. Introduction

Westernized diets, rich in refined sugar and saturated fat, are associated with several chronic diseases including cardiovascular disease and type 2 diabetes [1,2]. While discussion of Western diets often concentrates on these specific macronutrient types, this dietary pattern is also characterized by distinctive meals that may have acute, negative health effects that contribute to disease risk over time [3]. For example, the metabolic effects of “tailgating,” a social activity which involves high intake of alcohol and energy-dense foods for several hours, were recently examined. Over a five-hour period, tailgating increased insulin and triglycerides more than 7-fold and 3.5-fold, respectively, among other adverse metabolic changes [4]. Another study investigated ad libitum and maximal pizza consumption (similar to a buffet setting), finding that maximal consumption led to marked increases in serum insulin (~20-fold), glucose (~1.4-fold), and triglycerides (~2-fold) relative to baseline [5]. Though these studies offer insights into the effects of some meals consumed in Western cultures, other meals expose individuals to striking loads of refined sugar (~200 g in some cases) [6], the metabolic effects of which are not well understood.

Food offerings at commercial movie theater chains represent an opportunity to consume quantities of refined sugar far exceeding the daily recommended intake (i.e., ≤50 g per 2000 kcal) in one sitting [7]. Importantly, attending movies is a common social activity, evidenced by up to 41% of Americans seeing commercial movies on a regular basis in 2022 [8]. Although subtler than some meals consumed in movie theater settings, some studies have examined the cardiometabolic effects of sugar loads ranging from ~50 to 100 g. Beyond expected increases in circulating glucose and insulin, adverse changes in measures of vascular function and intestinal permeability (i.e., translocation of gut bacterial components into the blood that can drive inflammation) have been documented. For example, a 75 g glucose bolus decreased flow-mediated dilation (FMD), an indicator of vascular function, from baseline to one hour by 4.1% and 3.3% in apparently healthy and glucose-tolerant individuals, respectively [9,10]. Similarly, consuming a sugar-sweetened beverage (~72 g of unspecified sugar source) decreased FMD by 2.0% in apparently healthy individuals approximately 40 min after consuming the beverage [11]. With respect to intestinal permeability, acute increases in circulating lipopolysaccharide (LPS) have been reported two to three hours after drinking a sugar-sweetened beverage (110 g sucrose) and a mixture of glucose, fructose, and sucrose (57.5 g total sugar) in healthy adults [12,13].

While these data provide some insight into the acute effects of high sugar intake, common offerings in movie theater settings present the opportunity to consume significantly more refined sugar in a single sitting. Specifically, many cinemas have menu options to purchase large portions of sugar-sweetened beverage, candy, and popcorn (with added sodium and fat) in combination [6,14,15], which may lead to sugar consumption as high as ~200–250 g. Additionally, this scenario combines high sugar with nontrivial dietary sodium, which can also negatively influence certain cardiometabolic factors (e.g., FMD) [16]. Given their extreme nature and distinctive composition, the acute cardiometabolic effects of movie-theater-style meals warrant characterization. The present study was conducted to examine the cardiometabolic effects of consuming a movie-theater-style meal alone and following a high-fat meal. This design allowed us to (1) describe the independent effects of a movie theater meal and (2) characterize the response to the movie theater meal on the background of a Western-style meal, as many would do in their daily lives. In this context, we measured postprandial glycemia, lipids, vascular endothelial function, and indicators of intestinal permeability, which may be clinically relevant for predicting cardiometabolic diseases [17,18].

## 2. Materials and Methods

### 2.1. Participants

Participants (N = 10; 5M/5F; 18–45 years) were recruited through flyers, mass email, and word-of-mouth from Ball State University and the surrounding community. Participants did not have existing cardiometabolic conditions (e.g., cardiovascular disease, type 2 diabetes) or chronic inflammatory conditions (e.g., rheumatoid arthritis, inflammatory bowel disease), did not regularly use anti-inflammatory drugs (≤2 times per week), and had no history of glucose-lowering/lipid-lowering/illicit drug or tobacco use. Further, participants were not pregnant, expecting to become pregnant, or post-menopausal, and did not have a pacemaker. Participants provided written informed consent prior to participation, study procedures were carried out in accordance with the Declaration of Helsinki and approved by the Ball State University Institutional Review Board (IRBNet #2141491), and the study was pre-registered at clinicaltrials.gov (NCT06444984).

### 2.2. Study Overview and Design

**Figure 1:**
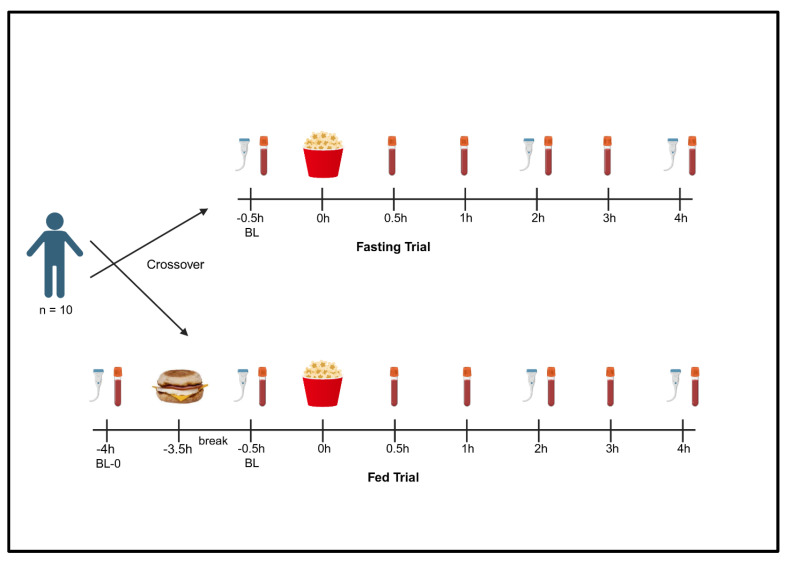
Study Design. Participants completed two meal trials in a random order: the Fasting Trial and the Fed Trial. At the Fasting Trial, serum markers and FMD were measured prior to movie theater meal consumption (BL). Additional blood samples were taken at 0.5, 1, 2, 3, and 4 h, and FMD at 2 and 4 h after the first bite. At the Fed Trial, serum markers and FMD were measured prior to consumption of a high fat meal (BL-0). After meal consumption, participants were allowed to leave for 3 h with instructions to remain sedentary and only consume water. Upon return, the Fed Trial procedures were identical to the Fasting Trial, starting with BL. Abbreviations: BL—baseline; FMD—flow-mediated dilation. Created in BioRender. Keirns, B. (2025) https://BioRender.com/z86j451 accessed on 15 February 2026.

Each participant completed a screening visit and two meal trials (Figure 1). At the screening visit, informed consent was obtained, and several one-time measurements were taken (i.e., anthropometrics, blood pressure). Next, participants completed two meal trials in the morning (6–10 AM start) on separate occasions in a randomized crossover design: a Fasting Trial to assess the independent effects of a movie-theater-style meal (i.e., meal consumed fasting) and a Fed Trial to determine the effects of the same movie-theater-style meal after first consuming a high-fat meal (see Meal Trials). The Fed Trial was designed to capture the response to a movie-theater-style meal in the postprandial state, specifically following a high-fat meal, as would be done by many in Western cultures. Meals rich in refined sugars are well known to impact measures of glycemia and many of our other outcomes (e.g., FMD) [9,10,19,20]. Given these established physiological effects of sugar consumption, we did not include an isocaloric, low-sugar control meal. Instead, we invested resources towards examining the effects of the movie theater meal alone, as well as the summative effect of a high-fat meal and the movie theater meal. The average washout period between trials was 10 days.

At the Fasting Trial, an intravenous catheter (IV) was inserted into an antecubital vein using a 21-guage needle, and a baseline blood sample was obtained (fasted ≥ 10 h). Following acclimation, a baseline assessment of endothelial-dependent vasodilation via brachial artery FMD was performed (see Flow-Mediated Dilation). Next, participants consumed a movie-theater-style meal, with their first bite recorded as time zero. Additional blood samples were collected at 0.5, 1, 2, 3, and 4 h, and FMD was repeated at 2 and 4 h, post-meal. The Fed Trial began with a baseline blood draw (fasted ≥ 10 h; single venipuncture) and FMD measurement to compare baseline data between trials. Participants then consumed a high-fat meal and were allowed to leave the laboratory for three hours with restrictions (i.e., remained sedentary, only consumed water). This approach was taken to reduce participant burden and because other studies have documented that metabolic factors are similar when participants remain in the laboratory and when they are allowed to leave under these restrictions [21]. Upon the participants’ return, procedures were identical to the Fasting Trial.

Participants were asked to maintain their habitual lifestyle behaviors prior to each study visit, which were assessed using 24 h food records and physical activity trackers (Vívosmart 5; Garmin, and/or smartphones). Food records were analyzed using the Nutrition Data System for Research Software (NDSR 2025; University of Minnesota). Step counts and minutes of moderate to vigorous physical activity were obtained from physical activity trackers. Participants also avoided anti-inflammatory medications for 72 h, as well as alcohol, exercise, and high-fat meals for 24 h prior to each trial.

### 2.3. Meal Trials

During both trials, participants consumed a movie-theater-style meal, consisting of popcorn with fat added by the manufacturer (42 g/1.5 oz/6 cups; Jolly Time Blast O Butter ™), candy (62 g/2.2 oz; Original Skittles ™), and a sugar-sweetened beverage (946 mL/32 fl oz; Original Taste Coca-Cola ™; Table 1). This meal was designed to imitate food combinations often consumed at commercial movie theaters (i.e., traditional foods, approximate serving sizes, rich in sugar and sodium) [6,14,15], but with serving sizes that would allow participants time to complete the meal prior to the 0.5 h blood draw. During the Fed Trial, participants consumed two pre-packaged breakfast sandwiches (Jimmy Dean sausage, egg and cheese biscuit; 820 total kcal; 54 g carbohydrate, 56 g total fat, 24 g protein, 1700 mg sodium) 3.5 h prior to eating the same movie-theater-style meal (first bite as time zero). All meals were consumed in ≤25 min.

### 2.4. Biochemical Analyses

At each time point, ~12 mL of whole blood was collected. ~2–3 mL of blood was transferred to a lithium heparin collection tube, inverted, and then used to measure total cholesterol (total-C), high-density lipoprotein cholesterol (HDL-C), triglycerides, alanine aminotransferase (ALT), aspartate aminotransferase (AST), and glucose (Piccolo Xpress; Abbott). Non-HDL-C, very low-density lipoprotein cholesterol (VLDL-C), and low-density lipoprotein cholesterol (LDL-C) were calculated. More specifically, LDL-C was calculated using the Friedewald equation (LDL-C = Total-C − HDL-C − (Triglycerides/5)). LDL-C was not measured at postprandial time points as it less accurate when triglycerides are raised. The remaining blood (~10 mL) was collected in serum separator tubes, allowed to clot for ~25 min, and centrifuged (15 min, 2500 RPM, 4 °C). Serum was then aliquoted and stored at −80 °C for future measurement of insulin (Eagle Biosciences, high sensitivity), lipopolysaccharide binding protein (LBP; Ray Biotech), and soluble cluster of differentiation 14 (sCD14; R&D Systems) with ELISAs. The ratio of LBP:sCD14 has been proposed as a strong indicator of postprandial intestinal permeability induced by high-fat meals and was calculated as an exploratory outcome [22,23]. For insulin, fasting serum samples were assayed undiluted and postprandial samples were diluted 1:20 in PBS + 5% BSA. Samples with optical densities above the highest standard when undiluted and at the 1:20 dilution were further diluted to 1:5 and 1:200 in PBS + 5% BSA, respectively, and re-assayed. Homeostatic model assessment for insulin resistance (HOMA-IR) was calculated using the original HOMA1 formula (HOMA1-IR = (fasting insulin [mU/L] × fasting glucose [mmol/L])/22.5) [24].

### 2.5. Body Composition Assessment

Participants underwent dual-energy x-ray absorptiometry (DXA) scans (Lunar iDXA; GE HealthCare, Chicago, IL, USA) to obtain body fat and lean mass metrics (i.e., total and percent body fat, total and percent lean mass, trunk fat). Waist circumference was measured at the level of the umbilicus using a Gulick tape.

### 2.6. Flow-Mediated Dilation

Brachial artery endothelial-dependent vasodilation was assessed using FMD. First, participants rested in a supine position for 10 min in a dark, quiet room to acclimate. A longitudinal image of the brachial artery was then obtained approximately three inches proximal to the antecubital fossa using a Doppler ultrasound and a 12 MHz probe (LOGIQ P9; GE HealthCare). A 10 min video recording was then initiated (USB Capture AiO; Magewell, Nanjing, Jiangsu, China). Following two minutes of baseline footage, a straight segmental wrist cuff (TMC7; Hokanson, Bellevue, WA, USA) placed two inches from the wrist was rapidly inflated to ~250 mmHg (E20 Rapid Cuff Inflator; Hokanson, Bellevue, WA, USA). After five minutes of occlusion, the cuff was released, and the hyperemic response was captured for three minutes. All videos were analyzed using an automatic wall-tracking software (Cardiovascular Suite FMD Studio version 5.0.0; Quipu). FMD data are expressed as absolute change in vessel diameter and the percent change in vessel diameter (all using maximal post-occlusion values relative to average baseline values).

### 2.7. Statistical Analyses

No studies to our knowledge have examined the acute effects of a movie-theater-style meal (rich in refined sugar and sodium) in any context, making a priori power analyses difficult for this descriptive study. Therefore, our statistical analyses plan and approximate target sample was modeled after Hengist et al. [5], who similarly described the postprandial responses to consuming two variations of a realistic meal (i.e., ad libitum versus maximal pizza consumption). First, all data were first checked for normality (skewness < 3, kurtosis < 10) [25]. Primary analyses consisted of mixed-model ANOVAs (meal trial × time), where significant meal effects indicated overall differences between meal conditions, irrespective of time; time effects denoted an effect of both meal trials, irrespective of the specific meal condition; and meal × time interactions indicated that Fasting and Fed Trials differed at ≥1 time point. Due to the requirement for balanced time points in mixed-model ANOVAs, the initial baseline measurement at the Fed trial (BL-0; Figure 1) was not included in these analyses. Instead, BL, 0.5, 1, 2, 3, and 4 h time points from each visit were used in ANOVAs. BL-0 data at the Fed Trial was used to verify that participants had similar fasting cardiometabolic factors upon reporting at each meal trial. Where applicable, ANOVAs were followed by Fisher’s post hoc test.

To compare the independent effects of the movie-theater-style meal versus the summative effects of the high-fat meal and movie-theater-style meal, change scores (∆) were also calculated. Specifically, the change between the first baseline obtained at each trial (BL Fasting and BL-0 Fed) and peak postprandial values were calculated for glucose, insulin, triglycerides, LBP, sCD14, and LBP:sCD14 at each meal trial, then compared using paired *t*-tests. Conversely, baseline to minimum postprandial values for HDL-C and FMD were calculated and compared in the same manner given previous literature demonstrating that these parameters typically decrease following acute sugar loads and/or high-fat meals [26,27]. One exception to these procedures was that the BL time point at Fed was not included as a potential maximum/minimum value, as both meals had not yet been consumed. Finally, physical activity and diet data prior to each trial, as well as true fasting values between each meal trial (i.e., BL-0 Fed versus BL Fasting; Figure 1) were compared using paired *t*-tests. Additionally, Pearson’s correlations were calculated to examine relationships between glycemia measures (i.e., glucose and ∆ insulin) and select body composition parameters at Fasting and Fed trials as an exploratory measure. Data were analyzed using SPSS 29 (IBM) and plotted in GraphPad Prism 10.2 (GraphPad Prism Inc., San Diego, CA, USA). Data are presented as mean ± SE and α = 0.05. Effect sizes for significant ANOVAs (η2) and paired *t*-tests (*d*) are reported in text.

## 3. Results

### 3.1. Participant Characteristics

Participant characteristics are displayed in Table 2. On average, participants had body mass indexes (BMIs) in the overweight class and had clinically normal glucose and lipid parameters. Of note, two participants had a fasting glucose of 101 mg/dL (suggestive of prediabetes), but fasting glucose was <100 mg/dL in all other participants. Fasting measures of glycemia, lipids of interest (i.e., triglycerides, HDL-C), FMD, and measures of intestinal permeability did not differ between each trial (i.e., BL-0 Fed versus BL Fasting; *p*’s ≥ 0.15; Table S1). Prior to each meal trial, energy intake, diet quality, and physical activity were similar (*p*’s ≥ 0.11, Table S1).

### 3.2. Glucose, Insulin, and Lipids

**Figure 2:**
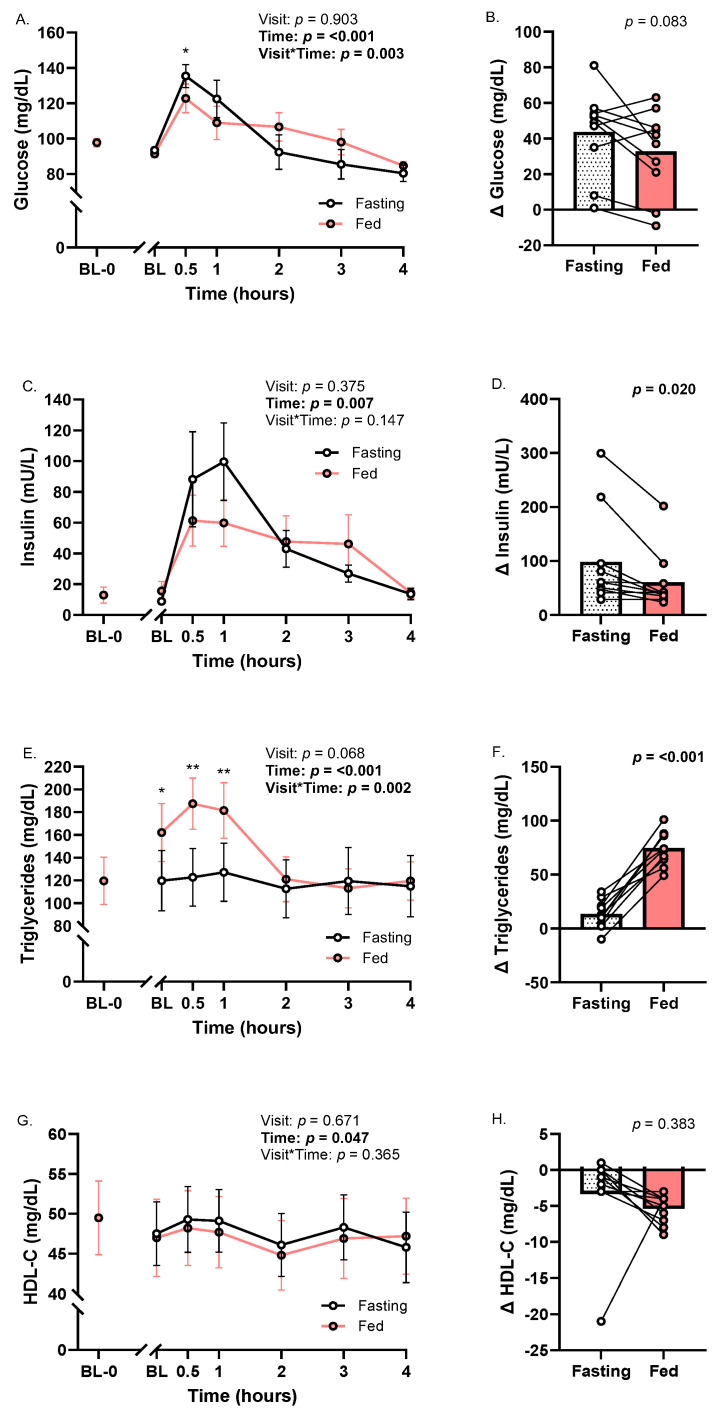
Glucose, insulin, and lipids at Fasting and Fed trials. (**A**) Glucose time course; (**B**) baseline to peak change in glucose; (**C**) insulin time course; (**D**) baseline to peak change in insulin; (**E**) triglyceride time course; (**F**) baseline to peak change in triglycerides; (**G**) HDL-C time course; and (**H**) baseline to minimum change in HDL-C. Abbreviation: HDL-C—high-density lipoprotein cholesterol. For the meal × time interaction effect, * *p* < 0.05 and ** *p* < 0.01. ANOVA data are represented as mean ± SE. Each Δ point represents the change from baseline to peak in an individual participant’s data.

Glucose and insulin increased with time across meal trials (*p*_time_’s < 0.01; η^2^’s ≥ 0.50; Figure 2A,C). Glucose was higher at the Fasting trial versus the Fed trial at 0.5h (*p*_meal×time_ < 0.01; η^2^ = 0.32; Figure 2A). Baseline to peak insulin (*p* < 0.05; *d* = 0.52; Figure 2D), but not glucose (*p* = 0.08; Figure 2B), was greater at Fasting compared to Fed. Measures of glycemia in response to the movie-theater-style meal at each trial varied widely across participants. Specifically, peak glucose ranged from 90 to 178 mg/dL at Fasting and 86 to 156 mg/dL at Fed (Figure 2A). Peak insulin ranged from 35.2 to 307.6 mU/L at Fasting and 28.3 to 212.0 mU/L at Fed (Figure 2C). The largest change in glucose (1.8-fold; 97 to 178 mg/dL) and insulin (~37-fold; 8.3 to 307.6 mU/L) took place at the Fasting trial (Figure 2B,D). Baseline to peak glucose and insulin were not correlated with body fat or lean mass parameters at either trial (*p*’s ≥ 0.11; Figure S1A–H).

Across trials, triglycerides increased (*p*_time_ < 0.001; Figure 2E) and HDL-C decreased (*p*_time_ < 0.05; η^2^ = 0.73; Figure 2G). Triglycerides were higher at BL, 0.5 h, and 1 h at Fed relative to Fasting (*p*_meal×time_ < 0.01; η^2^ = 0.54; Figure 2E). The change in triglycerides from baseline to peak was significantly higher at the Fed Trial than Fasting (*p* < 0.001; *d* = 0.49; Figure 2F).

### 3.3. Flow-Mediated Dilation

**Figure 3:**
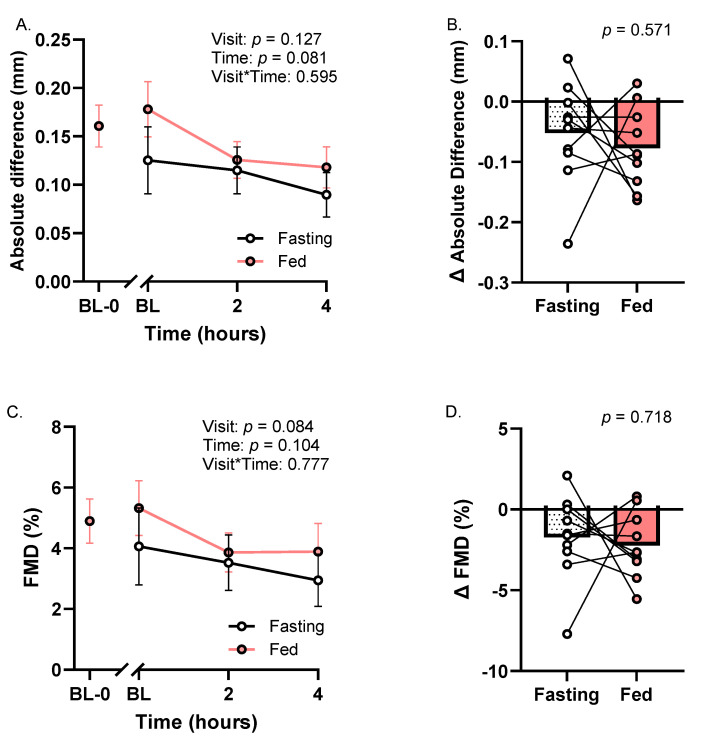
Vascular endothelial function at Fasting and Fed Trials. (**A**) Absolute difference time course; (**B**) baseline to minimum change in absolute difference; (**C**) FMD time course; and (**D**) baseline to minimum change in FMD. Abbreviation: FMD—flow-mediated dilation. ANOVA data are represented as mean ± SE. Each Δ point represents the change from baseline to peak in individual participant data.

Overall, there were no significant meal, time, or meal × time interaction effects for the absolute difference in vessel diameter or FMD percent (*p*’s ≥ 0.08; Figure 3A,C). However, time was associated with large effect sizes for decreased absolute difference (partial η^2^ = 0.22; Figure 3A) and FMD percent (partial η^2^ = 0.22; Figure 3C). Baseline to minimum changes in absolute difference and FMD percent were not different across meal trials (Figure 3B,D).

### 3.4. Indicators of Intestinal Permeability

**Figure 4:**
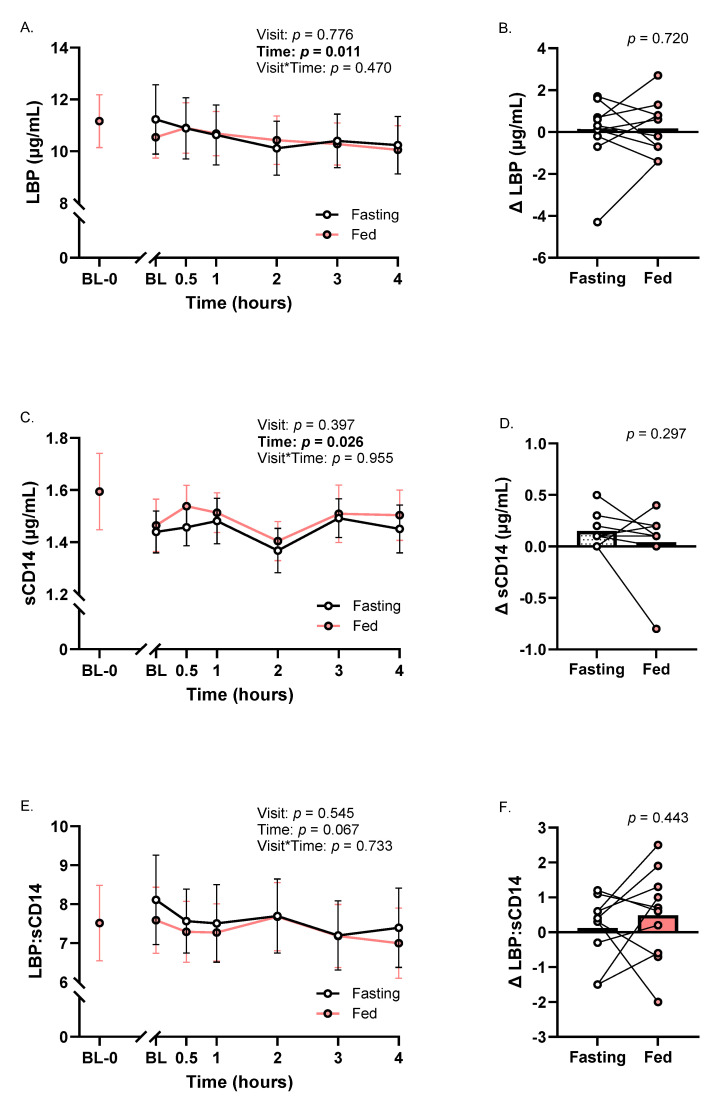
Biomarkers of intestinal permeability of Fasting and Fed Trials. (**A**) LBP time course; (**B**) baseline to maximum change in LBP; (**C**) sCD14 time course; (**D**) baseline to maximum change in sCD14; (**E**) LBP:sCD14 time course; and (**F**) baseline to maximum change in LBP:sCD14. Abbreviations: LBP—lipopolysaccharide binding protein; sCD14—soluble cluster of differentiation 14. ANOVA data are represented as mean ± SE. Each Δ point represents the change from baseline to peak in an individual’s participant data.

No meal or meal × time effects were observed for LBP and sCD14 (Figure 4A,C). A time effect for circulating LBP was observed (*p*_time_ < 0.05; η^2^ = 0.27; Figure 4A). Somewhat similarly, sCD14 was lower at 2 h than most other time points (*p*_time_ < 0.05; η^2^ = 0.24; Figure 4C). Changes in LBP and sCD14 from baseline to peak were not different between meal trials (Figure 4B,D). LBP:sCD14 was unaltered across all analyses (*p*’s > 0.06; Figure 4E,F).

## 4. Discussion

In the present study, we aimed to characterize the independent cardiometabolic effects of a movie-theater-style meal alone and following high-fat meal consumption. This work was motivated by recent research that has examined other distinctive and extreme meals commonly consumed in Westernized countries (e.g., tailgating) [4,5]. In our study, glucose and insulin increased during both meal conditions, as expected. However, notable inter-individual variability was observed in measures of glycemia. Though vascular function was not impaired, we did observe large effect sizes consistent with FMD decreasing with time under both meal conditions. With respect to gut integrity, we noted decreases in LBP and sCD14 across trials.

Pronounced glucose and insulin responses to meals rich in added sugar are linked to oxidative stress, low-grade inflammation, impaired vascular properties, and future risk for cardiometabolic conditions (e.g., type 2 diabetes, cardiovascular disease) in healthy populations [10,28,29,30]. For these reasons, we examined whether individuals free of chronic disease experienced marked increases in glucose and insulin following consumption of movie-theater-style meals. As expected, glucose and insulin increased after both meal trials. Perhaps more notable was the variability in these responses across participants. For example, glucose remained ˂95 mg/dL throughout both meal trials in one participant, while glucose in another participant nearly doubled (92 mg/dL to 178 mg/dL). However, it is worth noting that the latter had a HOMA-IR > 5 and would be considered insulin-resistant by some groups [31]. With respect to circulating insulin, variability was even more extreme. Insulin in one participant remained ≤37 mU/L throughout both meal trials (5-fold increase from baseline). Conversely, insulin reached 308 mU/L (37-fold increase from baseline) in another participant at the Fasting trial. We anticipated that measurements of glucose and insulin would inversely and positively correlate with lean mass and body fat parameters, respectively. However, these relationships were not observed in our study, which may be due to our modest sample size. Finally, we observed that glucose was higher at 0.5 h post-movie-theater-style meal at the Fasting compared to Fed trial. These data are consistent with an observation described as the “second meal effect,” (i.e., lower glucose response to identical meals if another meal was eaten previously) [32,33].

Although participants consumed a relatively extreme sugar bolus in the present study, these data can still be interpreted in the context of everyday variation in glycemia markers and eating patterns. In a NHANES sample, random plasma glucose ≥ 140 mg/dL was associated with 8.4-fold increased risk for undiagnosed prediabetes or type 2 diabetes [17]. In our sample, seven participants achieved this threshold at one or more meal trials. It is likely that these responses reflect the sugar load consumed and not the presence of prediabetes/diabetes, but this comparison shows the magnitude of the glucose response to the movie-theater-style meal. Our data can also be compared to other studies examining realistic Western-style meals recently examined. Mean insulin achieved at the Fed Trial was comparable to that of tailgating, but the insulin response at the Fasting Trial was nearly 1.5-fold that of tailgating [4]. However, maximal pizza consumption led to an insulin response that was 1.5-fold that of the Fasting Trial [5]. From a glucose standpoint, there were no notable descriptive differences between our work and these other meal studies.

Beyond directly impacting metabolic markers, high sugar and sodium intake have individually been associated with impaired endothelium-dependent vasodilation measured by FMD [9,10,11,16]. Indeed, previous work documented that FMD decreased between 40 and 60 min after glucose boluses of 72–75 g [9,11], while another noted a trending, but non-significant, decrease in FMD in glucose-tolerant individuals 60 min after consuming 75 g of glucose [10]. With respect to sodium, acute intake of 1500 mg decreased FMD [16]. In the present study, we observed that, across both trials, FMD (absolute difference and percent change) did not decrease over time (*p*’s ≥ 0.08). This partial contrast to the existing literature may be attributable to our selection of later time points (i.e., 2 and 4 h in our study rather than ≤1 h), differences in glucose delivery (i.e., mixed meal versus a bolus), and/or lower sodium content of the movie-theater-style meal. Nonetheless, it is worth considering that we observed a large effect size associated with descriptive decreases in FMD, suggesting that an adverse effect on FMD may be observed in a larger sample. Future work examining the combination of high sugar and salt in realistic meals would ideally address this limitation (i.e., with a larger, more diverse sample) and include additional measures of vascular health (e.g., arterial stiffness measures).

Intestinal permeability and subsequent increases in circulating endotoxin have been linked to increased risk for cardiometabolic disease, arguably by contributing to chronic inflammation [34,35,36]. Previous studies reported that ~60 g and 110 g glucose loads increased circulating LPS and toll-like receptor 2 ligands (suggesting translocation of bacterial products from the gut) [12,13]. In contrast to these data, we observed a decrease in LBP and sCD14 with time across both meal trials. These findings are unexpected but could be related to our selection of secondary markers of LPS exposure. In other words, it is possible that LPS increased but was not accompanied by increases in LBP and sCD14 in the time course measured herein. That said, these indirect markers of endotoxemia were chosen given concerns around environmental contamination when assessing LPS. Somewhat similar to our work, Hawkesworth et al. [37] reported that fasting endotoxin-core IgM antibodies (another secondary marker of LPS exposure) were unexpectedly lower in women with obesity and type 2 diabetes versus lean women. The authors speculated that lower endotoxin-core IgM antibodies in this context reflected increased antibody turnover following LPS neutralization. Perhaps something similar occurred in our study, although this cannot be determined. In any case, these data further suggest that measures of intestinal permeability are influenced by high-sugar meals.

Although this is the first study of our knowledge to examine the acute consequences of movie-theater-style eating, there are some limitations to note. First, we acknowledge that the present work is an acute study. Future work should investigate long term implications of chronic movie theater meal consumption. Additionally, the average BMI of participants was in the overweight range and data were collected in the United States with meals based on American movie theater meals and eating context. These factors limit the generalizability of findings to other BMI categories, dietary patterns, or metabolic backgrounds. Future research might include larger and more diverse populations with culturally representative meals. We also acknowledge it would have been ideal to measure hemoglobin A1c to provide more information on participants’ long-term glucose control. Third, we acknowledge that some individuals may consume movie theater meals over longer periods of time (i.e., >20 min). That said, our approach allowed us to maintain consistency between participants and ensured that meals were completed prior to the 0.5 h time point. Additionally, we could have included a low-sugar, high-carbohydrate control meal to provide more certainty that our observed effects were due to the movie theater meal’s sugar content, rather than total carbohydrates or energy. However, we opted to focus on the independent and post-high-fat meal effects of the movie theater meal because of the large literature base showing refined sugar strongly influences postprandial glucose and insulin [19,20]. Finally, we acknowledge our modest sample size obtained through convenience sampling, which may have inadvertently led to selection bias. Despite these limitations, our study provides insight into the effects of the distinctive meal experience at a movie theater through a controlled, yet ecologically valid approach. We examined the independent effects of the movie-theater-style meal as well as the additive effects after a high-fat meal, which may be more similar to realistic conditions.

## 5. Conclusions

Overall, we conclude that acute consumption of a movie-theater-style meal negatively affected several cardiometabolic factors regardless of whether eaten alone or following a high-fat meal, although the glycemic and insulinemic response varied widely across individuals. Future research should explore the mechanisms underlying the variability in response to movie-theater-style meal consumption (e.g., differences in body fat storage patterns, insulin sensitivity).

## Figures and Tables

**Table 1:** Nutrient composition of a movie-theater-style meal.

	Energy (kcal)	CHO (g)	Sugar (g)	Fat (g)	SFA (g)	Protein (g)	Sodium (mg)
Popcorn	270	24	0	18	9	4	570
Skittles ^TM^	242	57	46	2	2	0	10
Coca-Cola ^TM^	372	104	104	0	0	0	120
Totals	884	185	150	20	11	4	700

Abbreviations: CHO—carbohydrate; SFA—saturated fatty acid.

**Table 2:** Participant characteristics.

Participant Characteristics	All (N = 10)
**General/Body Composition**	
Age (years)	29 ± 3
Sex (M/F)	5/5
BMI (kg/m^2^)	28.7 ± 2.6
Waist circumference (cm)	92.5 ± 7.4
Body Fat (%)	32.5 ± 3.5
Lean Mass (%)	63.9 ± 3.4
Trunk Fat (g)	15,529 ± 3631
Systolic BP (mmHg)	119 ± 6
Diastolic BP (mmHg)	76 ± 2
**Fasting Metabolic Parameters**	
Glucose (mg/dL)	96 ± 2
Total-C (mg/dL)	173 ± 7
HDL-C (mg/dL)	51 ± 4
LDL-C (mg/dL)	99 ± 7
VLDL-C (mg/dL)	23.6 ± 4.7
Non-HDL-C (mg/dL)	123 ± 9
Triglycerides (mg/dL)	117 ± 24
ALT (U/L)	31 ± 3
AST (U/L)	29 ± 2
Insulin (mU/L)	8.8 ± 2.0
HOMA-IR	2.1 ± 0.5

Abbreviations: BMI—body mass index; BP—blood pressure; Total-C—total cholesterol; HDL-C—high-density lipoprotein cholesterol; LDL-C—low-density lipoprotein cholesterol; VLDL-C—very low-density lipoprotein cholesterol; ALT—alanine aminotransferase; AST—aspartate aminotransferase; and HOMA-IR—homeostatic model assessment for insulin resistance. Data are represented as mean ± SE.

## Data Availability

The raw data supporting the conclusions of this article will be made available by the authors on request.

## Supplementary Materials

The following supporting information can be downloaded at: https://www.mdpi.com/article/10.3390/metabo16020139/s1, Table S1: Participant Characteristics; Figure S1: Correlations Between Change in Insulin and Glucose and Body Composition Parameters at Fasting and Fed Trials.
