# Supplementary material for: The “Movie Theater” Study: Acute Cardiometabolic Effects of a Cinema-Style Meal
Source: Metabolites. 2026 Feb 18;16(2):139. doi: 10.3390/metabo16020139 (PMC12943263; doi:10.3390/metabo16020139)
Supplement: Supplementary file 1 [file metabolites-16-00139-s001.zip › metabolites-4113629-supplementary.pdf]

	Fasting Trial	Fed Trial	*p*-value
**Diet**			
Energy (kcal)	2173 ± 200	1869 ± 111	0.114
Carbohydrate (g)	245.8 ± 39.6	211.2 ± 20.4	0.285
Fat (g)	86.9 ± 9.6	72.0 ± 9.9	0.132
Saturated fat (g)	24.7 ± 3.6	20.6 ± 3.0	0.417
Protein (g)	88.3 ± 15.6	77.3 ± 11.6	0.675
HEI (a.u., 0-100)	50 ± 5	56 ± 5	0.353
**Physical Activity**			
Steps/day	9007 ± 1327	8497 ± 1281	0.549
MVPA (min)	17 ± 8	12 ± 6	0.322
**Fasting Metabolic Parameters**			
Glucose (mg/dL)	93.5 ± 1.7	94.65 ± 1.0	0.610
Insulin (mU/L)	8.8 ± 2.0	12.8 ± 5.3	0.268
HOMA-IR	2.1 ± 0.5	3.0 ± 1.3	0.276
Triglycerides (mg/dL)	119.7 ± 26.6	119.5 ± 20.9	0.988
HDL-C (mg/dL)	47.5 ± 4.0	49.5 ± 4.6	0.368
**Fasting Vascular Endothelial Function**			
FMD%	4.1 ± 1.3	4.9 ± 0.7	0.542
Absolute difference (mm)	0.13 ± 0.03	0.16 ± 0.02	0.423
**Fasting Intestinal Permeability Markers**			
LBP (μg/mL)	11.2 ± 1.3	11.2 ± 1.0	0.921
sCD14 (μg/mL)	1.4 ± 0.1	1.6 ± 0.1	0.158

Table S1. Participant Characteristics. Abbreviations: HEI—healthy eating index; MVPA—moderate to vigorous physical activity; HOMA-IR—homeostasis model for insulin resistance; HDL-C—high-density lipoprotein cholesterol; FMD—flow-mediated dilation; LBP—lipopolysaccharide binding protein; sCD14—soluble cluster of differentiation 14. Data are represented as mean ± SE.

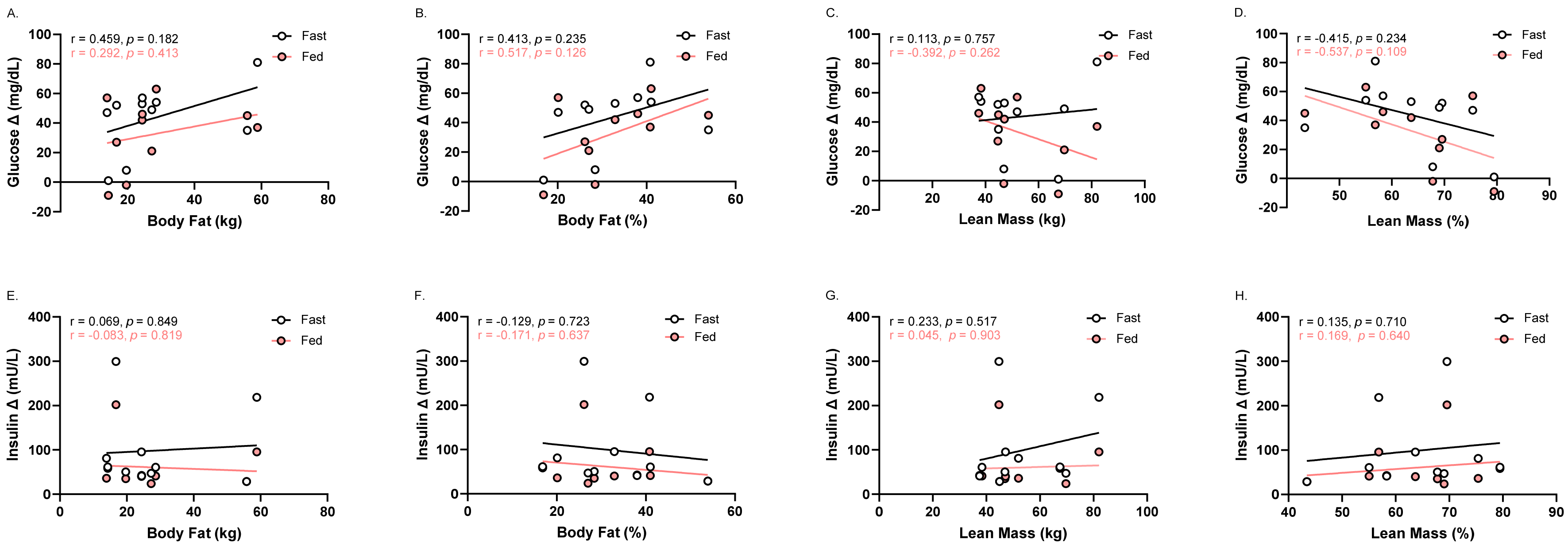

Figure S1. Correlations Between Change in Insulin and Glucose and Body Composition Parameters at Fasting and Fed Trials. (A) Change in glucose and absolute body fat; (B) Change in glucose and percent body fat; (C) Change in glucose and absolute lean mass; (D) Change in glucose and percent lean mass; (E) Change in insulin and absolute body fat; (F) Change in insulin and percent body fat; (G) Change in insulin and absolute lean mass; (H) Change in insulin and percent lean mass. Each data point represents individual participant data.
